# Molecular Hydrogen Therapy—A Review on Clinical Studies and Outcomes

**DOI:** 10.3390/molecules28237785

**Published:** 2023-11-26

**Authors:** Hennie Marie Johnsen, Marianne Hiorth, Jo Klaveness

**Affiliations:** 1 Department of Pharmacy, University of Oslo, Sem Sælands Vei 3, 0371 Oslo, Norway; 2 Nacamed AS, Oslo Science Park, Guastadalléen 21, 0349 Oslo, Norway

**Keywords:** medical gas, hydrogen therapy, molecular hydrogen, clinical trials, antioxidant, human studies

## Abstract

With its antioxidant properties, hydrogen gas (H_2_) has been evaluated in vitro, in animal studies and in human studies for a broad range of therapeutic indications. A simple search of “hydrogen gas” in various medical databases resulted in more than 2000 publications related to hydrogen gas as a potential new drug substance. A parallel search in clinical trial registers also generated many hits, reflecting the diversity in ongoing clinical trials involving hydrogen therapy. This review aims to assess and discuss the current findings about hydrogen therapy in the 81 identified clinical trials and 64 scientific publications on human studies. Positive indications have been found in major disease areas including cardiovascular diseases, cancer, respiratory diseases, central nervous system disorders, infections and many more. The available administration methods, which can pose challenges due to hydrogens’ explosive hazards and low solubility, as well as possible future innovative technologies to mitigate these challenges, have been reviewed. Finally, an elaboration to discuss the findings is included with the aim of addressing the following questions: will hydrogen gas be a new drug substance in future clinical practice? If so, what might be the administration form and the clinical indications?

## 1. Introduction to Hydrogen Therapy

The most frequently used medical gases include medical air, oxygen, nitrogen, nitrous oxide and carbon dioxide, and hydrogen gas (H_2_) is a promising newcomer with unique antioxidant properties. Demonstrated to selectively counteract deleterious reactive oxygen species (ROS), such as the hydroxyl radical, H_2_ can maintain tissue homeostasis and can be more clinically useful than strong antioxidants that indiscriminately neutralize both beneficial and harmful ROS species [1]. Initially introduced in diving gas around 1970, H_2_ was thought to be non-toxic and biologically inert [2]. The interest in using H_2_ for disease treatment has increased since the antioxidant properties were unveiled in 2007 [3]. Since then, over 2000 scientific publications have elucidated its therapeutic promise from in vitro, in vivo animal and human studies.

The expanding body of literature substantiates therapeutic effects of H_2_ and clinical trials have been conducted within major disease areas like cardiovascular, respiratory and cancer, with a focus on diseases associated with the accumulation of ROS. Yet, translation into standard clinical practice presents challenges. One challenge is administration of a decent H_2_ dose due to its low water solubility and its explosive properties when mixed with oxygen gas (O_2_). Drinking H_2_-saturated water has been reported to be a common and feasible method but H_2_ has a low water solubility of 1.57 mg/L (1.57 ppm), corresponding to approximately 19 mL/L at standard ambient temperature and pressure (SATP) conditions. Therefore, to obtain several milligrams of H_2_ per day, the ingestion of several liters of saturated hydrogen water per day is required [4]. The injection of H_2_-rich saline is also limited by its low aqueous solubility and can only be performed with proper equipment, which is typically performed in selected clinics. The explosive/flammable danger limit of H_2_ in O_2_ mixtures is about 4% [2] and, surprisingly, administration by inhalation is performed both below and above this limit. H_2_ is the smallest molecule with a molecular weight of only 2 Da, a kinetic diameter of 289 pm [5] and a density of about 0.089 g/L [6]. Therefore, it can easily permeate biological membranes and diffuse throughout the body, including to the brain. This has been shown for all the abovementioned administration methods in biodistribution studies in rats and pigs [7,8].

Commercially, H_2_ is predominantly generated by electrolysis for industrial and green fuel applications. H_2_-enriched water, specialized machines for H_2_ generation and products for making H_2_-rich water like magnesium effervescent tablets are already being sold around the world, marketed with health benefits. The global hydrogen generation market size was valued at USD 155.35 billion in 2022 [9]. Yet, its medical utility remains a fraction of this figure.

Despite compelling evidence from animal and human studies, H_2_ is yet to gain universal acceptance in clinical settings. Potential hindrances encompass possible lack of regulatory documentation studies of efficacy and safety, administration intricacies, and safety concerns tied to its explosive tendencies in the presence of oxygen. Based on the increasing number of scientific publications within the field, the authors have asked the following questions:

- Will H_2_ emerge as a novel drug in clinical practice?
- Which administration form optimizes the efficacy of H_2_?
- What are the potential clinical applications for H_2_?

These three questions form the scientific basis for the current review publication.

## 2. Methods

Published scientific literature related to H_2_ therapy was identified by specific searches using the terms “hydrogen therapy”, “medical use of hydrogen” and “hydrogen gas” in combination with “human study” and “clinical trial”, in several readily searchable publication databases including PubMed. Scientific papers written in a different language than English and those studying human effects of alkaline ionized water were excluded. An overview of the clinical trials was gathered from www.clinicaltrils.gov (accessed on 30 September 2023), the international clinical trials register by U.S. Department of Health and Human Services (National Institutes of Health, National Library of Medicine), and www.umin.ac.jp/ctr/ (accessed on 30 September 2023), the Japanese preregistration system for approved clinical trials (UMIN, University Hospital Medical Information Network). The relevant clinical trials were found by searching for “hydrogen”, “hydrogen gas” and “H2” (trials about “hydrogen peroxide” and “hydrogen breath test” were excluded). Publications related to the clinical trials were also included in the present review. This research resulted in 47 and 34 clinical trials, from cliniclatrials.gov and UMIN, respectively, and 64 publications on human studies (in addition to several relevant animal and in vitro studies). A review article published in 2015 made a summary of the clinical trials up until that year, and the present summary therefore mainly focuses on the clinical data produced in the period after [10].

This review publication is sectioned as follows. Section 3 of this review presents and discusses the quantitative data of clinical trials and publications related to H_2_ therapy. Section 4 discusses safety of H_2_ administration, which is the first important topic upon clinical translation of a novel drug. Section 5 includes a tabular overview of the clinical trials registered at clinicaltrials.gov and UMIN and discusses the findings there. Section 6 qualitatively investigates the data from published reports after human studies, including clinical trials, by describing the study methodology and the main findings. These results formed the basis for the discussion in Section 7.

## 3. Quantitative Overview of Clinical Trials and Scientific Publications

**Figure 1:**
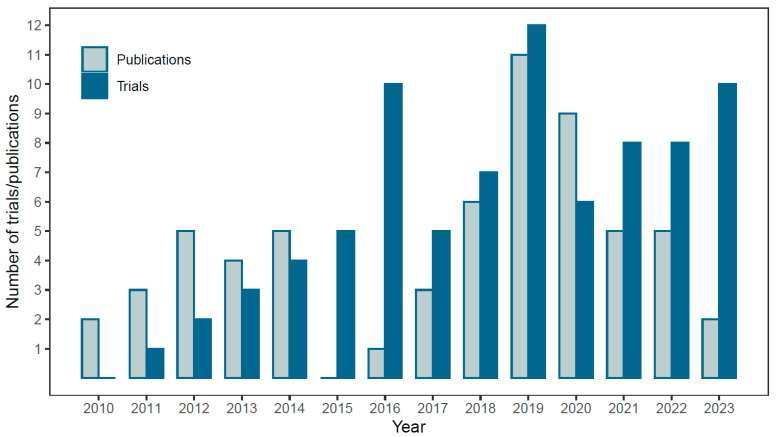
The number of clinical trials and scientific publications about hydrogen therapy in humans, sorted after publication year from 2010 to 2023 (per August 2023).

A quantitative overview of the number of clinical trials and scientific publications about H_2_ therapy in humans are shown as a function of the publication year, and the first publication date for the clinical trials, in Figure 1. The number of clinical trials has increased from one registered in 2011, five in 2015, with a boost to ten in 2016 and 12 in 2019, to 6–10 each year in the period 2020–2023. The number of scientific publications does not follow the same trend and has been more stable over the period 2012–2022 (data only until August 2023), with an average of approximately five publications per year, and a maximum of 11 publications in 2019. The increased publication activity during 2019–2020 can possibly be explained by the high number of clinical trials initiated in 2016 and the sudden interest in hydrogen therapy in COVID-19 studies. Therefore, the high number of clinical trials registered from 2019 to 2023 might result in a subsequent upswing in the number of scientific publications in the near future.

**Figure 2:**
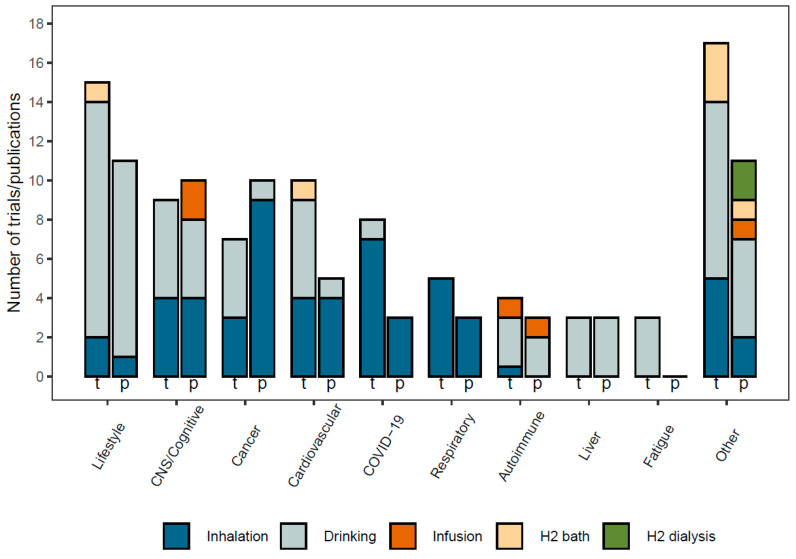
Registered clinical trials (t) and scientific publications (p) about hydrogen therapy, sorted by disease indication and administration method.

The publications and clinical trials sorted after the indication area and administration route are shown in Figure 2. The major disease areas globally are represented in this graph, including central nervous system (CNS), cancer, cardiovascular and respiratory. Lifestyle-related conditions also have a high number of both trials and publications, and this area is becoming increasingly important with a global population on average increasing in age and weight. Inhalation is the dominating administration method, followed by drinking H_2_-enriched water, infusion of H_2_-enriched saline. H_2_-bath/eye-drops and H_2_-dialysis have also been used in a few trials.

## 4. Human Safety of H_2_ Administration

Ensuring safety is the foremost consideration when the aim is clinical translation of a new drug substance. As opposed to other medical gases like carbon dioxide, nitric oxide, and hydrogen sulfide, H_2_ does not bind to hemoglobin in the bloodstream, thus H_2_ will not induce heme-related toxic effects. Extensive animal studies have shown that H_2_ administration is safe, yet comprehensive documentation of human safety across diverse administration methods remains to be demonstrated. A mixture of H_2_:O_2_ or H_2_:O_2_:He gas has been employed by deep-sea divers to prevent decompression sickness for a long time, therefore also confirming human safety of H_2_ inhalation. Two studies from 1988 and 1994 concluded that breathing mixtures comprising 49–56% H_2_ during dives to 450–500 m below sea level can alleviate some symptoms of the high-pressure nervous syndrome and confirmed the safety in use [11,12]. Notably, recent clinical studies aim to document human safety of H_2_ therapy, for instance the clinical studies NCT04046211 and UMIN000013221 (Table 1 and Table 2). One study involving ten cerebral ischemia patients demonstrated that inhaling 3% H_2_ for 30 min did not change any physiological parameters, reaffirming safety. Additionally, an increase in H_2_ blood levels, equivalent to findings in animal studies, was observed. However, some inconsistencies in the H_2_ levels between the individuals were also observed [13]. The high number of the clinical trials conducted (Table 1 and Table 2) underpins the safety and the nearly absent toxicity of H_2_ administration by drinking H_2_-saturated water, inhaling H_2_ gas of different levels, injection of H_2_-rich saline and other methods such as topical application and dialysis. Very few adverse reactions from human H_2_ consumption have emerged across the reported clinical studies, and all trials have concluded that H_2_ administration is safe for humans. Still, concerns about H_2_ flammability warrant continued consideration.

## 5. Overview of Registered Clinical Trials with H_2_ Administration

Once the safety of H_2_ administration is confirmed, the subsequent objective is to document the therapeutic efficacy of a novel drug substance, a prerequisite for its integration into everyday clinical practice. Unlike conventional medications typically evaluated for specific diseases, H_2_, as an antioxidant, can offer versatile application across various medical conditions. An overview of the 47 clinical trials registered at clinicaltrials.gov and 34 clinical trials registered in the UMIN database investigating hydrogen therapy is tabulated in Table 1 and Table 2, respectively (as of August 2023). The tables are sorted after disease area and administration method. Many of the listed trials are within large disease groups like cardiovascular, CNS, respiratory and cancer.

These encompass the major healthcare challenges of today demanding innovative pharmaceutical interventions. Successful outcomes of these trials bear the potential to significantly contribute to addressing the foremost sources of disease and mortality within the present global landscape [14].

A few different technological solutions have been employed to facilitate H_2_ administration in the clinical trials tabulated above. This includes inhalation machines that aim to make the mixture of H_2_ and O_2_ safer and more manageable. A hydrogen-oxygen generator device developed by Shanghai Asclepius Meditech Inc. (Shanghai, China) has been used for the inhalation of a gas mixture of 66% H_2_ and 33% O_2_ (3 L/min, model AMS-H-03). This technology was used in several trials for COVID-19, cancer, respiratory and cardiovascular diseases, where many of which Shanghai Asclepius Meditec Inc. was the responsible party or sponsor. Similarly, Qingdao Haizhisheng Corp. (Qingdao, China) has introduced a hydrogen generator (model HZS-2700A) delivering a flow rate of 2 L/min, which was employed in a type 2 diabetes trial. Additionally, investigations have explored the inhalation of lower H_2_ concentrations, typically around 2%. A Japanese company, Doctors Man Co., Ltd. (Yokohama, Japan), provides products for H_2_ administration such as H_2_ water generators and gas inhalers.

Several suppliers of products related to H_2_-rich water consumption have also been identified in the compiled clinical trials. Notably, electrolyzed water or magnesium (Mg) tablets that produce H_2_ from reaction with water, said to give concentrations of 2 ppm (2 mg/L) H_2_ and above, are widely used with intake of typically 0.5–1 L/day. DrinkHRW (New Westminster, Canada) is one supplier of Mg tablets and Nihon Trim Co., Ltd. (Osaka, Japan) is a supplier of electrolyzed water. Oral administration is one of the easiest to implement for self-administration at home. Moreover, suppliers of ready-to-drink H_2_-rich water used in clinical trials include Rejuvenation, HRW Natural Health Products Inc. (New Westminster, BC, Canada), Aquastamina-R, Nutristamina (Ostrava, Czech Republic and Melodian Co., Ltd. (Osaka, Japan). Some claim to have achieved H_2_ concentrations of over 7 ppm (7 mg/L) in 500 mL of water and 15 ppm (15 mg/L) in 250 mL of water [15]. Another company, HoHo Biotech (Taipei, Taiwan), has developed capsules of porous coral material that they claim can absorb and carry hydrogen. Administered orally, the nanoscale carrier can release the enclosed H_2_ within the body [16]. This technology has been applied in trials involving patients with rheumatologic conditions [17].

The commercial interest in developing technologies for H_2_ administration and the geographical diversity of clinical trials show an excitement for H_2_ therapy spreading across continents. Predominantly, these trials are conducted in Europe, USA and Asia. A major part of the reported clinical trials has been conducted in Japan (Table 2); however, China also emerges as a center for many of the trials, particularly those related to COVID-19. Notably, the exercise-related studies are conducted in Serbia and Czech Republic.

## 6. Efficacy Results of H_2_ Administration in Human Studies

An overview of the conducted and to-be conducted clinical trials in a certain therapeutic field, such as the one provided above, can say something about the interest in the field, however it does not consider the outcomes. The efficacy of H_2_ administration has been reported in at least 64 referee reviewed scientific publications that are qualitatively discussed below; many but not all are results of the clinical trials listed above. In the following presentation, the articles are categorized into sections according to the primary disease areas investigated. These categories encompass cardiovascular, cancer, respiratory, CNS, infections, lifestyle-related and other diseases, and quantitatively discuss the findings from human studies.

### 6.1. Cardiovascular Diseases

Cardiovascular diseases are the leading cause of deaths worldwide [14], and by such are in need of new therapies. Several clinical studies have concluded that H_2_ inhalation or drinking can improve the outcomes of cardiovascular diseases, most in combination with standard treatments. An early phase/initial clinical trial assessed the effect of inhaling 2% H_2_ gas, in combination with target temperature management, in a total of ten patients with post-cardiac arrest syndrome. H_2_ inhalation was found to maintain a favorable neurological outcome in patients, even though improvements were not statistically significant. However, the 90-day survival rate was significantly better in the H_2_-group, compared to control [18]. In another study involving five post-cardiac arrest syndrome patients given the same treatment as above, arterial H_2_ concentration was measurable and indications of reduced amounts of oxidative stress and cytokine levels were found [19]. A slightly larger study including 20 patients found health-promoting effects of hydrogen therapy for adverse left ventricular remodeling after percutaneous coronary intervention for patients after myocardial infarction. At the 6-month follow-up after initiating treatment with 1.3% H_2_ inhalation, the improvement in the left ventricular stroke volume index and ejection fraction was numerically greater than in the control group, even though the latter was not significant. Indications were given for initiating a large-scale efficacy trial [20]. H_2_ has also been shown as a useful modulator of blood vessel function. Flow-mediated dilation was significantly improved for patients who drank H_2_-rich water (7 ppm = 7 mg/L), compared to placebo [21]. These studies show that the inhalation of a low concentration H_2_-containing gas and drinking of H_2_-rich water can be useful in treating cardiac and vascular conditions, respectively.

### 6.2. Cancer

**Figure 3:**
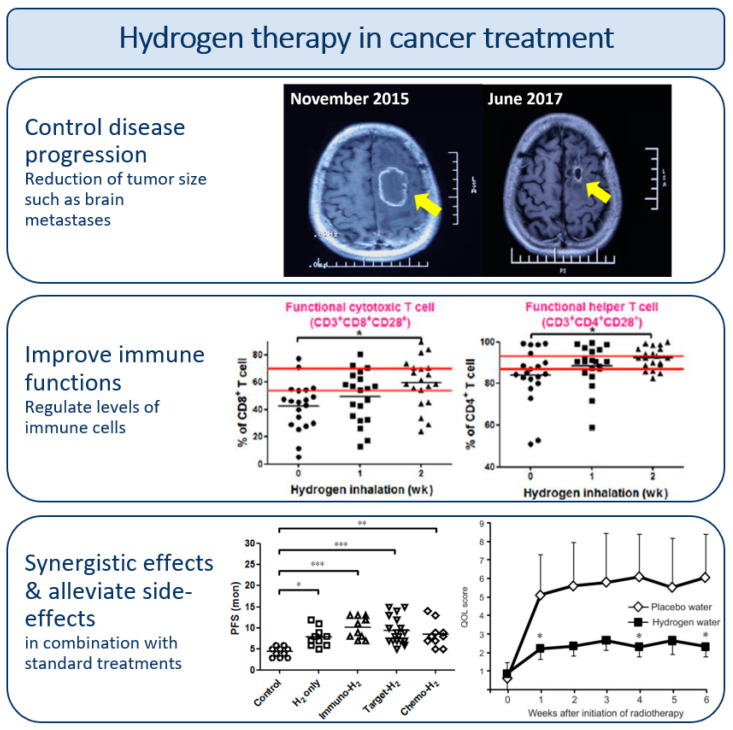
Schematic overview of the effects of hydrogen therapy in cancer treatment. The top images show MR imaging of brain metastasis (arrow) before (**left**) and after (**right**) H_2_ treatment, adapted from [24]; OncoTargets and Therapy 2019:12 11145-11151—originally published by, adapted and used with permission from Dove Medical Press Ltd., Macclesfield, UK. The middle figures show alterations in cytotoxic and helper T cell levels in cancer patients after H_2_ treatment, adapted from [31]. The parallel red lines show the normal range of cell levels and the black short lines show the measured average values at each time point. The bottom left figure shows number of months (mon) with progression-free survival (PFS) of lung cancer patients receiving no treatment (control), H_2_ therapy (H_2_ only), or combined treatments with H_2_ and immunotherapy (immuno), targeted therapy (target) or chemotherapy (chemo). The black short lines show the average PFS values for each treatment. Adapted from [25]. The bottom right figure shows the quality of life (QOL) scores of patients treated with radiotherapy for liver tumors, with or without H_2_ water, adapted from [27]. A higher score reflects more symptoms and lower QOL. * *p* < 0.05, ** *p* < 0.01, *** *p* < 0.001, for all figures.

Cancer is the second leading cause of death in the world today [14]. H_2_ has been administered to cancer patients with the purpose of controlling tumor progression, for combination treatment and to alleviate side-effects and adverse events of standard cancer treatment in a variety of cancer diseases. Additionally, H_2_ administration has been shown to promote antitumor immune responses. Figure 3 summarizes the benefits of hydrogen therapy in cancer patients.

Two separate case studies with cancer patients with multiple metastases have shown tremendous effects of H_2_ inhalation therapy. One of the patients suffered from recurrent gallbladder carcinoma and was given daily H_2_ inhalation therapy. In the first month, the tumors continued to progress following a gradual decrease in tumor size and tumor marker levels that eventually returned to normal. After around two and a half months, the patient could resume normal life and survival was reported still after 10 months. A pseudo-progressive remission after H_2_ therapy was observed, which may resemble the pattern that occurs following PD-1 antibody treatment. This suggests that H_2_ can affect the immune system [22,23]. The other case report was a non-small cell lung cancer patient that underwent H_2_ gas inhalation as monotherapy after oral and surgical treatments had stabilized the first lesions. The brain metastases reduced in size after four months of H_2_ treatment and completely disappeared after one year. The liver and lung metastases were also stabilized after one year, and survival was lengthened [24]. These case studies are tremendous observations and show that H_2_ might elicit significant control of tumors after standard cancer treatments have failed. However, it does not empirically prove that H_2_ has medical effects in cancer patients and for this we will need statistical grounds from systematic clinical trials.

One clinical study including 58 patients with advanced non-small cell lung cancer reported in 2020 that H_2_ therapy was able to relieve pulmonary symptoms compared to a control group that received no treatment. The hydrogen-group was administered H_2_ by inhalation for 4–5 h per day for 5 months. The same hydrogen-treatment was also given to non-small cell lung cancer patients in combination with either chemotherapy, targeted therapy or immunotherapy. After 16 months, the progression-free survival was higher for the hydrogen-only treatment group, and significantly higher for all the combination-treatment groups, as compared to the control group [25]. Another trial reported benefits of H_2_ treatment in 42 lung cancer patients treated with nivolumab. Significantly longer overall survival was found for the combination treatment with H_2_ gas compared to those treated with nivolumab only. It was suggested that the two therapeutic agents might exhibit synergistic effects as mitochondrial activators [26]. It is reasonable to treat lung cancer with H_2_ inhalation to target the disease site, even though systemic effects have been observed. Other cancer types that have shown positive outcomes from H_2_ therapy by inhalation or drinking include liver, nasopharyngeal and colorectal cancer [27,28,29] as well as head and neck cancer (see Table 1).

Interesting immune-modulating effects of H_2_ administration have been observed in cancer patients. A high proportion of immune cells of the type CD8+ T cells expressing the programmed cell death protein-1 (PD-1) is often seen in cancer patients and can be associated with poor cancer prognosis. PD-1 is an immune checkpoint receptor that guards against autoimmunity and is often involved in immunotherapy treatment (PD-1 inhibitors) as it makes the immune system “oversee” cancer cells. The administration of H_2_ has been shown to reduce the proportion of PD-1+ CD8+ T cells in the blood of cancer patients. This has been observed in both late-stage colorectal carcinoma and lung cancer patients in separate clinical trials and H_2_ has shown to improve cancer prognosis in both patient groups [26,29]. Furthermore, H_2_ can improve the efficacy of nivolumab treatment in cancer patients with high levels of PD-1+ CD8+ T cells, which previously had a poor response to nivolumab [26]. A significant decrease in the proportion of PD-1+ CD8+ T cells after H_2_ inhalation treatment was also seen in a case study of a gallbladder carcinoma patient [22]. The loss of immunological activity in the CD8+ T cells may be due to mitochondrial dysfunction, in which H_2_ is found to be an important player. The colorectal carcinoma patients treated with a combined therapy of H_2_ and nivolumab showed a significantly longer overall survival than the patients who were treated with nivolumab alone [30]. Another study found that patients with non-small cell lung cancer that inhaled H_2_:O_2_ (2:1) without other treatments resulted in reestablishment of normal levels of six cell subsets involved in our immune system, including cytotoxic T cells, T helper cells and natural killer cells [31]. From these appealing results, the use of H_2_ could be interesting in combination with immunotherapy for modulating immune reactions towards cancer cells.

In addition to the direct effects of H_2_ therapy in controlling tumor progression, several cell and animal studies [32,33,34,35] have shown that H_2_ can be effective in alleviating side-effects without reducing anti-tumor activity of standard cancer treatment. The array of side-effects may be experienced as devastating for the quality of life of cancer patients and survivors alike. Therefore, clinical studies have investigated the effect of H_2_ on chemo- and radiotherapy induced injuries. A study with 134 colorectal cancer patients found that hydrogen-rich water can alleviate mFOLFOX6 chemotherapy-induced liver injuries [36]. Non-small cell lung cancer patients undergoing chemotherapy, targeted therapy or immunotherapy treatment experienced a decrease in adverse events after H_2_ administration, and for some it even disappeared [25].

Radiotherapy is an important treatment for several cancer diseases. Side-effects of radiotherapy are often associated with increased generation of ROS that can potentially be reduced with H_2_ treatment. One study tested whether six weeks of drinking H_2_-rich water improved the quality of life for 49 patients with liver tumors who received radiotherapy. It was shown that H_2_-rich water consumption reduced the biological response to oxidative stress induced by radiation without comprising the anti-tumor effects of radiotherapy. Quality of life-scores were significantly improved for patients receiving H_2_-therapy in combination with radiotherapy compared to patients receiving placebo water [27]. An observational study found that H_2_ inhalation treatment can significantly alleviate radiotherapy-induced bone marrow damage, such as the reducing effects of white blood cells and platelets, without compromising the anti-tumor effects [37]. Adverse reactions like difficulty of swallowing, brain injury and hearing loss are often experienced after radiotherapy for nasopharyngeal cancer. Three nasopharyngeal cancer patients were reported to have moderate-extremely severe hearing loss and needed hearing aids after radiotherapy. The patients received H_2_ administered by inhalation for four hours every day (2:1 = H_2_:O_2_). After 1–2 months, the patient’s binaural hearing had improved considerably, and one of the patients no longer needed a hearing aid [28]. These findings are promising, however, more research is needed to reach definitive conclusions.

### 6.3. Respiratory Diseases

When listing the leading causes of death worldwide, respiratory conditions come third [14], and they are still in need of new pharmaceutical solutions. A gas mixture of helium and oxygen has been used for decades to treat obstructive pulmonary disease for its lower density, higher viscosity and reduced airway resistance compared to the conventional nitrogen-oxygen mixtures [38]. Using oxygen-hydrogen mixtures can have similar effects, in addition to the therapeutic antioxidant effects of H_2_.

In 2020, a study that examined the acute effects of inhalation of H_2_-containing gas found that inflammatory status in asthma and COPD patients was attenuated by the treatment. The trial included 20 asthma and COPD patients that inhaled a 2.4% H_2_-containing steam mixed gas for a single inhalation period of 45 min. This treatment significantly decreased inflammation markers like monocyte chemotactic protein 1, IL-4 and IL-6 levels in both COPD and asthma patients [39]. In 2021, a larger clinical trial was conducted with 108 patients with acute exacerbation of COPD receiving either H_2_:O_2_ or O_2_ therapy. They found superior improvement in symptoms in the H_2_:O_2_ group with significant results in certain test scores [40], showing that COPD is an interesting indication for H_2_ therapy. The authors have not been successful in identifying more extensive studies of hydrogen therapy for asthma, despite the positive findings. Another clinical study from 2018 evaluated the efficacy and safety of breathing H_2_ in acute severe tracheal stenosis patients. Thirty-five patients were administered H_2_:O_2_ gas mixture (2:1) for 15 min and 120 min (6 L/min) in two consecutive breathing steps. All the measured endpoint parameters except vital signs were improved after inhaling H_2_, including inspiratory effort as assessed by diaphragm electromyography (EMGdi), transdiaphragmatic pressure (Pdi), Borg score, and impulse oscillometry (IOS) [41]. These clinical studies show that H_2_ inhalation is a promising treatment option for respiratory diseases, which is a suitable administration method both for targeting the diseased tissue and for convenience as breathing devices are often used by respiratory disease patients. Additionally, the inhalation of H_2_ has also been tested in COVID-19 infected patients for treating respiratory symptoms, with very promising results (see Section 6.5).

### 6.4. Central Nervous System Diseases

Conditions affecting the central nervous system (CNS) represent one of the major public health challenges today. Dementia and other diseases causing cognitive decline are correlated with an aging population which is an increasing problem and new medications to treat these diseases are badly needed. Molecular hydrogen is the smallest molecule, and its very small size and nonpolar nature makes it highly diffusible. It can even pass through the blood-brain barrier which is a major obstacle for medical treatment of the brain [8].

A few clinical studies have investigated the effects of hydrogen therapy on acute damage to the brain caused by cardiac occurrences, all of them showing benefits of H_2_ administration. In one study, thirty-seven patients with poor-grade subarachnoid hemorrhage were given a H_2_-rich solution by infusion for 14 days together with intracisternal magnesium sulfate infusion, or only intracisternal magnesium sulfate infusion. Incidences of cerebral vasospasm and delayed cerebral ischemia were significantly lower in the treatment groups and H_2_-therapy had additional effects by decreasing serum malondialdehyde, a marker of oxidative stress, reduced biomarkers for neuronal damage and physical therapy improvement by the Barthel Index [42]. In another study, 25 patients with cerebral infarction were given H_2_ inhalation (3% H_2_ gas) treatment for one hour twice a day. H_2_ inhalation, compared to control, gave significant effects on lower relative MRI signal, indicating less severe infarction site, neurological improvement seen by NIHSS scores and improved Barthel Index scores [43]. Improved MRI indices for brainstem infarction patients were also observed after intravenous administration of H_2_-rich saline, in combination with edaravone. The results were better for the combined treatment than for edaravone treatment alone [44]. For giving direct access to the brain via the blood-brain barrier, H_2_-rich saline is more often administered intravenously in clinical trials involving brain disorders as compared to drinking and inhalation that is more widely used for other conditions. This can also be due to convenience because critically ill patients are hospitalized and are typically prepared for IV administration of various drugs. However, other administration routes might also be useful for treating critically ill patients. A large-scale trial with 73 patients at 15 hospitals in Japan studied the effect of H_2_ inhalation on neurological outcomes for patients with brain ischemia during post-cardiac arrest. The patients were randomly assigned to receive O_2_ with or without 2% H_2_ for 18 h. An increase was seen in the primary neurological outcome, however, this was not statistically significant. The reported statistical significance in the secondary outcomes, on the other hand, including an increase in 90-day survival, suggests that H_2_ inhalation may have therapeutic benefits without neurological deficits [45].

Clinical trials with hydrogen therapy have also been conducted with patients with cognitive disorders including Alzheimer’s and Parkinson’s disease, as reduced neurodegeneration have been seen from several animal studies with H_2_ [46,47,48]. In one human study, 73 patients with mild cognitive impairment drank 300 mL of H_2_-enriched water or placebo water every day for a year and their Alzheimer’s Disease Assessment Scale-cognitive subscale (ADAS-cog) scores were then determined. The study found that carriers of the apolipoprotein E4 (APOE4) genotype significantly improved their total ADAS-cog scores after drinking H_2_ water when compared to the control group. This indicates that genetic variations can affect how subjects respond to hydrogen treatment. However, there was no significant difference between the H_2_ and the control groups in ADAS-cog scores after 1 year [49]. Another study involving eight patients diagnosed with Alzheimer’s disease suggests that 3% H_2_ inhalation can relieve symptoms and has disease-modifying effects. Improvements in ADAS-cog scores and integrity of neurons, seen by diffusion tensor imaging (DTI), were significant during the 6-month follow-up but non-significant after one year, compared to untreated patients [50].

A trial tested H_2_ inhalation in 20 Parkinson’s disease patients. The inhalation of either low dose H_2_ of ~1.3% in air or placebo for 10 min twice a day for 4 weeks did not result in any beneficial effects. However, another interesting finding of increased 8-hydroxy-2′-deoxyguanosine (8-OHdG, an indicator of cellular oxidative stress) and other reported stress responses, suggest that beneficial effects of hydrogen therapy are partly or largely mediated by hormetic mechanisms [51]. A larger-scale similar study following 178 patients over 72 weeks revealed no disease improvement by oral H_2_ consumption (1 L/day) in patients with Parkinson’s disease [52]. Even though beneficial effects of hydrogen therapy were not found in this large-scale study of Parkinson’s disease, combinatory treatment might show more benefits. A pilot study including 18 patients with Parkinson’s disease found significant improvements in the total Unified Parkinson’s Disease Rating Scale (UPDRS) scores compared with the baseline after combined treatment of daily drinking H_2_-saturated water and photobiomodulation (PBM) over a two-week period. The report attributes the positive effects to “PBM targeting the brainstem may facilitate neuronal activity, and the concomitant H_2_ may clear additional ROS produced by PBM” [53]. A randomized, double-blind pilot study performed in 2013 including 18 Parkinson’s disease patients treated with levodopa showed that drinking 1 L of H_2_ water every day for 48 weeks significantly improved the UPDRS scores [54]. The improvements can, however, be attributed to the alleviation of side-effects of the other drug taken in combination, as observed for cancer treatments.

### 6.5. Infections

A flourishment of H_2_ therapy occurred as the pandemic of the Coronavirus disease 2019 (COVID-19) spread globally. H_2_ inhalation has been used to treat respiratory-related COVID-19 symptoms in several clinical studies, primarily for the same reasons as its use in other respiratory conditions, namely a reduction in gas density and airflow resistance. However, the studies do not rule out possible beneficial antioxidant effects of H_2_.

**Figure 4:**
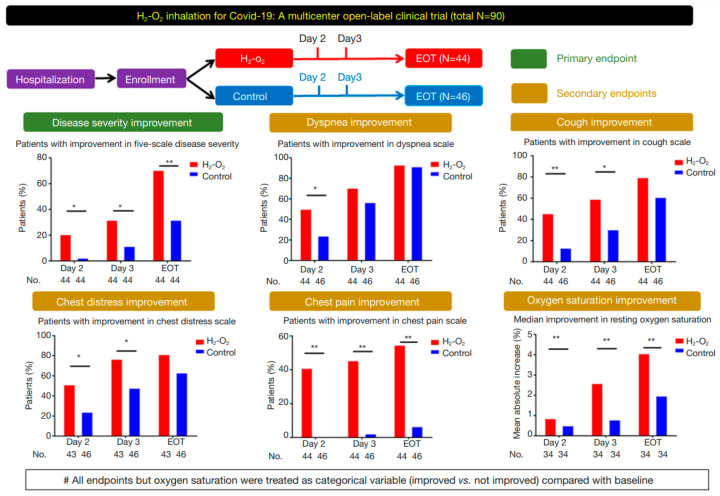
Trial overview and results from a successful COVID-19 study, showing all end-point parameters were positive after H_2_-O_2_ inhalation compared to control. * *p* < 0.05, ** *p* < 0.01, EOT = end-of-treatment. Reproduced with permission from [55], Journal of Thoracic Disease; published by AME Publishing Company, Hong Kong, 2020.

A pioneering clinical study conducted with 100 patients at seven different Chinese hospitals found exclusively positive results of continuous inhalation of H_2_:O_2_ 2:1 gas mixture (3 L/min) in all the end-point parameters, compared to standard oxygen treatment, as shown in Figure 4. The primary endpoint was disease severity, and the secondary endpoints were dyspnea, cough, chest distress, chest pain and oxygen saturation, and all were significantly improved after just two days of treatment compared to control [55]. These excellent outcomes resulted in the inclusion of this specific treatment (H_2_:O_2_ inhalation 2:1) as a recommendation by the China National Health Commission in the “Chinese Clinical Guidance for COVID-19 Pneumonia Diagnosis and Treatment (7th edition)” [56], next to standard oxygen therapy. This practice does not seem to have attracted attention globally in the combat against COVID-19, and additional clinical trials have been performed (see Table 1). A study published in 2022 investigated the use of H_2_ therapy in rehabilitation of 50 acute post-COVID-19 patients. They found significant improvements in 6-min walking test distance, forced vital capacity and expiratory volume when administered H_2_ by inhalation. The protocol described at-home inhalation of 100% H_2_ at low flow rate (250 mL/min) for 60 min twice a day for two weeks [57].

A retrospective study, also published in 2022, of medical records of twelve COVID-19 patients that were subject to H_2_:O_2_ therapy by inhalation found suppression of inflammatory responses, compared to standard treatment. For the H_2_ group, the results showed a significant decrease in neutrophil percentage and the concentration of C-reactive protein [58]. In summary, H_2_ inhalation can be beneficial for COVID-19 patients for at least three different reasons; (i) a reduction of ROS levels, (ii) reduced airflow resistance for eased respiratory conditions and (iii) a reduction in inflammatory responses. All the positive results of both improved physical and respiratory function in COVID-19 patients have demonstrated the clinical usability of this novel therapeutic strategy.

Hydrogen therapy in the treatment of other infections has shown health-promoting effects in various animal models including sepsis and periodontitis, a serious gum infection, by reducing inflammation and sepsis-induced injuries [59,60,61]. No in-human studies have investigated the effect of H_2_ on sepsis, but one clinical study found elevated oxidative stress levels in patients with chronic hepatitis B. Sixty patients were included, some were administered 1.2–1.8 L/day of H_2_-rich water, and some were given routine treatment for 6 consecutive weeks. A significant reduction in ROS was seen after H_2_ therapy, compared to the control group. Improved liver function and hepatitis B DNA levels were comparable after both H_2_ and control treatment, and the report suggests that longer-term studies might be needed to confirm the physiological effects of H_2_ on the reduction of oxidative stress [62]. To summarize, the treatment of infection-related symptoms using H_2_ has shown positive outcomes, but there has been no documentation that the underlying infection itself is affected by H_2_ therapy.

### 6.6. Lifestyle-Related Conditions and Exercise

Hydrogen therapy has also been used in the treatment of lifestyle-related and metabolic conditions, which is an increasing problem with an increasingly heavier population. Many studies also point to the beneficial effects of H_2_ therapy in relation to exercise and sports-related injuries, including administration forms like H_2_ inhalation, drinking of H_2_-rich water and topical H_2_ application. A pilot study found that oral intake of H_2_-rich water prevented an elevation of blood lactate and decreased muscle fatigue during heavy exercise in ten male elite soccer players and suggests that it can be a suitable means of hydration for athletes [63]. Another study involving eight male cyclists found similar results, oral intake of H_2_-saturated water improved performance in intermittent cycling exercises when the duration was longer than 30 min (anaerobic) [64]. A clinical trial involving ten men and ten women found an increase in peak running velocity up to 4.2% in a running time-to-exhaustion test after seven days of inhaling 4% H_2_ for 20 min each day [65]. One study showed that drinking H_2_-rich water before and after strenuous exercise reduced the exercise-induced increase in ROS levels in eight male volunteers. This might help prevent accumulative muscular fatigue even though exercise performances were not significantly different over the two 3-day consecutive exercise tests, compared to placebo water [66]. A larger-scale study investigated the effects of drinking H_2_-enriched water right before cycle ergometer exercise sessions in healthy non-trained (*n* = 99) and trained (*n* = 60) participants. They found that H_2_-water, compared to placebo water, enhanced endurance and relieved psychometric fatigue as measured by maximal oxygen consumption and Borg’s scale and visual analogue scales, respectively [67]. Additionally, healing of acute sports-related soft tissue injuries can be positively impacted by both oral and topical H_2_ treatment for 2 weeks, next to standard-care [68]. Flipping the coin from high-level exercise, H_2_ therapy has also been studied for managing lifestyle-related disease states. Typically drinking 1–2 L of H_2_-rich water daily is shown to reduce ROS levels and contribute to body fat reduction in overweight people [69], as well as managing lipid and glucose metabolism in patients with diabetes type 2 [70] and metabolic syndrome [71,72,73,74].

### 6.7. Other Diseases and Conditions

Outside the major disease areas discussed herein, several clinical studies investigating H_2_ on other indications related to high oxidative stress levels have been reported. Rheumatoid arthritis is an autoimmune disease characterized by chronic inflammation and the destruction of bone and cartilage. Elevated hydroxyl radical levels are thought to be involved in pathogenesis, for which H_2_ therapy can have a selective scavenging effect. Both drinking and injection of H_2_-enriched water and saline have been tested for the treatment of rheumatoid arthritis in separate clinical trials. In one study, twenty patients drank 530 mL of H_2_-rich water/day for 4 weeks in two separate periods. In another study, 24 patients were randomly assigned either H_2_-saline or placebo-saline which was administered intravenously by drop infusion of 500 mL daily for 5 days. The results of both studies showed that H_2_ significantly reduced biomarkers for oxidative stress (for instance urinary 8-hydroxydeoxyguanine) and disease activity score in 28 joints (DAS28), by C-reactive protein levels. This was accompanied by significant improvements in rheumatoid arthritis symptoms [75,76]. Chronic graft-versus-host-disease can also be considered an autoimmune disease. A longer-term study over 12 months found therapeutic effects of daily drinking H_2_-rich water at a dose of 12 mL/kg. Out of the 24 patients enrolled, 18 had an objective response as measured in seven domains (skin, mouth, gastrointestinal, liver, eyes, lungs and joints and fascia) using the National Institutes of Health (NIH) Consensus form for measuring therapy response for chronic graft-versus-host-disease. The survival time and the survival rate at 4 years was significantly prolonged in the response group, compared to the nonresponse group. There is currently no standard therapy for chronic graft-versus-host-disease patients refractory or dependent to corticosteroid treatment, and therefore H_2_ can serve as a good option [77].

Another worldwide health issue, without any approved medications, is non-alcoholic fatty liver disease (NAFLD), causing hepatic dysfunction. Metabolic impairment plays a major role in NAFLD pathogenesis, so pharmaceuticals that advance lipid and glucose metabolism are of interest for treating this disease. Hydrogen possibly has these properties, as seen from the results of the clinical trials with diabetes and metabolic syndrome patients. NAFLD can also be associated with inflammation, excess oxidative stress and aberrant cellular signaling. Two separate clinical trials studied the effect of oral intake of H_2_-rich water at 1 L/day. One of them included 12 subjects and found that after one month, the H_2_ therapy significantly reduced liver fat accumulation, as compared to placebo, with no significant differences in body weight and composition [78]. The second study involved 30 subjects and lasted for 2 months and found non-significant decreases in levels of NAFLD disease markers, as well as weight and body mass index. Interestingly, non-significant increase tendencies of oxidative stress markers (8-hydroxy-2′-deoxyguanosine and malondialdehyde) were also found, which was explained by the hormetic effects of H_2_ occurring prior to the significant clinical improvements over the longer-term [79]. Another study tested 13-week inhalation of H_2_:O_2_ (2:1) gas mixture at rate of 3 L/min for 1 h/day in 43 NAFLD patients with moderate-severe cases. Improvements in serum lipid and liver enzymes and significantly improved liver fat content was found. Additional studies in mice models revealed that this effect of H_2_ was possibly caused by promoting hepatic autophagy [80].

Some specific conditions have been studied for the effect of H_2_-enrichment in other administration forms such as solutions for dialysis and via the eyes. One study demonstrated significant effects of using H_2_ solutions during cataract surgery (phacoemulsification) to restore vision. The procedure employs ultrasound which produces free radicals that can potentially be neutralized with H_2_-enriched solutions. Thirty-two patients with cataracts in both eyes were treated with the conventional method on one eye and using H_2_-enriched solution on the other eye. Reduction rates of endothelial cell density, the primary endpoint, were significantly smaller in the H_2_ group at all the measured time points [81].

Hemodialysis patients experiencing chronic inflammation have poor prognoses and therapeutic approaches are limited. Therefore, the anti-inflammatory properties of H_2_ can be utilized in the dialysis solution for these patients. Two studies performed by the same research group, published in 2010 and 2018, investigated the effects of H_2_-infused dialysate, with H_2_ from water electrolysis, in the treatment of hemodialysis patients. In the 2010 study, 21 patients were switched to H_2_-enriched dialysis solution for 6 months, resulting in significant decreases in systolic blood pressure compared to before the test period. A significant decrease in plasma inflammation markers were also identified [82]. The 2018 study was longer-term and had a larger test group of 161 and 148 patients administered H_2_-enriched and conventional dialysis solutions, respectively, over a 3.3-year observation period. Reductions in post-dialysis hypertension were found and multivariate analysis revealed H_2_-enriched dialysis as an independent significant factor for the primary endpoint, which was a composite of all-cause mortality and development of non-lethal cardio-cerebrovascular events [83]. In both studies, H_2_ caused minimal changes in dialysis parameters but the positive results including improved blood pressure control and ameliorated inflammatory reactions could improve the prognosis of chronic hemodialysis patients.

Others have studied H_2_ therapy for the treatment of various diseases involving inflammation and pain. Four patients with acute erythematous skin diseases with fever and/or pain experienced significant improvement in symptoms, that did not recur, after infusion with H_2_-enriched fluid (500 mL/day for 3 days) [84]. Twenty-eight patients with interstitial cystitis/painful bladder syndrome were given H_2_-rich water or placebo water for 8 weeks. Even though H_2_-rich water was extremely effective in improving the bladder pain score in 11% of the patients, the improvements were not significantly different from that of placebo [85]. H_2_-enriched water has also been tested on patients with mitochondrial and inflammatory myopathies. Observations included improved mitochondrial dysfunction in mitochondrial myopathy patients and inflammatory processes in patients with polymyositis/dermatomyositis. Significant improvements in certain disease markers were observed, even though the improvement in clinical symptoms was not significant. Two different therapy schemes were followed, where 14 patients drank 1 L/day for 12 weeks and 22 patients drank 0.5 L/day for 8 weeks. Less prominent effects were seen for the lower amount scheme, illustrating a dose-dependent response of H_2_ therapy [86].

## 7. Summary and Discussion

Qualitative and quantitative analysis of the current data related to human use of H_2_ therapy has been conducted herein, encompassing over 64 clinical studies and 81 registered clinical trials. All the studies have unequivocally confirmed the safety and legitimacy of H_2_ in human consumption in all the administration methods tested, and therefore set the foundation for clinical trials for a variety of indications. The high numbers of ongoing clinical trials registered over the recent years underscores the global effort for rapid introduction of H_2_ therapy in various disease areas spanning chronic to acute stages. Phase 2 and 3 clinical trials are ongoing, yet the area is still in its infancy.

The exploration of H_2_ consumption’s efficacy has encompassed several interesting indications spanning the major disease areas. Noteworthy positive outcomes have emerged among patients suffering from myocardial infarction, an array of cancers (lungs, liver, colorectal and gallbladder), asthma, COPD, brain ischemia, COVID-19, rheumatoid arthritis, non-alcoholic fatty liver disease and in hemodialysis patients. Also, lifestyle-related indications have shown promising results, including type 2 diabetes, obesity and exercise performance. Given the molecular effects of mitigating the most damaging reactive oxygen species, H_2_ should be most effective in treating diseases correlated with high levels of oxidative stress. Moreover, most disease states involve dysregulation of ROS and therefore it can be a diverse medication. Promisingly, several clinical studies have identified the reduction of oxidative stress biomarkers, confirming the mechanism of action. Despite these robust indicators, the road to clinical translation might prove more intricate compared to conventional new drugs that are developed by pharmaceutical companies and tailored to specific indications.

Respiratory health emerges as a promising arena for hydrogen therapy, particularly in the context of respiratory diseases and COVID-19 infections. This is evidenced by the integration of H_2_ inhalation in the China National Health Commission’s recommendation for COVID-19 treatment. While the reduction in airway resistance due to the low density of H_2_ is a possible factor, its antioxidant potential can have additional benefits.

Cancer remains an interesting field for H_2_ therapy, offering not only direct effects on cancer progression but also demonstrating relief from side-effects of standard treatments. Furthermore, synergistic effects from combining H_2_ with conventional treatments, such as chemotherapy and radiotherapy, hold promise for optimizing therapeutic outcomes and enhancing overall quality of life. The observed immuno-modulating effects of H_2_ have the potential to complement immunotherapy, which further augments its therapeutic potential. Hydrogen therapy has been used to complement conventional therapies in clinical trials including cardiac arrest, brainstem infarction and Parkinson’s disease patients, in addition to cancer. Due to low side-effects and potential beneficial effects, the health risks can be greater for not using H_2_ in combination with standard treatments across a broad spectrum of conditions from cancer to sports-related injuries.

Ideally, H_2_ therapy would be beneficial for diseases that currently have no treatment options. Cognitive disorders, which are increasing concurrently with the elderly population, exemplify such unmet needs. While small-scale human trials offered hope for therapeutic effects of H_2_ in Parkinson’s and Alzheimer’s disease, large-scale studies have found no significant positive results. Use in other diseases without standard medical regimes, however, such as non-alcoholic fatty liver disease or hemodialysis, shows promise. In summation, the landscape of H_2_ therapy’s clinical utility is multifaceted and promises both challenges and opportunities, yet its integration with conventional treatments warrants further exploration.

Undeniably, the administration method plays a major role in disease treatment. Oral consumption of H_2_-rich water, suitable for most diseases, and inhalation of H_2_ gas mixtures, used prominently for respiratory symptoms, are emerging as the predominant methods. Low-dose (4% or below) H_2_ inhalation is often used to mitigate the explosion risk. Injection is most relevant for hospitalized patients and the use of H_2_-enriched dialysis solutions and water baths show potential in suitable disease contexts. Another factor in the therapeutic equation is the dose of H_2_ administered. Inhalation enables sustained, safe high-dose administration, while drinking may necessitate consumption of either large amounts of saturated water or high concentration of supersaturated water in order to obtain an optimal H_2_ dose.

Innovations within micro- and nanomaterial drugs for in vivo H_2_ generation are promising for advancements of the field [87,88]. These new drugs can allow for feasible oral administration of a tablet that will dissolve in the gastrointestinal (GI) system and, in reaction with the water available, produce H_2_ that can freely diffuse throughout the body. Some candidate materials include magnesium (Mg), magnesium hydride (MgH_2_) and silicon (Si) that can react with water to produce H_2_ in the following reactions:

Mg + 2H_2_O → Mg(OH)_2_ + H_2_ (1)

MgH_2_ + 2H_2_O → Mg(OH)_2_ + 2H_2_ (2)

Si + 2H_2_O → SiO_2_ + 2H_2_ (3)

Reaction (1) follows the same principle as the Mg effervescent tablets for H_2_-rich water generation. The theoretical capacity (at SATP: 1 bar and 25 °C) is 1.02 L/g and animal studies have shown that Mg-containing materials can produce H_2_ for treatment of osteoarthritis and gastric cancer [89,90,91]. MgH_2_ can produce more H_2_, up to 1.89 L/g, and oral MgH_2_ administration to mice has been shown to alter gene expression to enhance fatty acid metabolism [92]. The use of MgH_2_ in vase water is also shown to be applicable in postharvest flower preservation by extending the vase life of cut roses [93]. Si has a theoretical H_2_ generation capacity of 1.77 L/g (reaction 3) and Si nanoparticles have been shown as an effective candidate for H_2_ generation in simulated GI environments [94]. Animal studies have confirmed positive effects of orally administered H_2_-generating Si in several disease models including pneumonitis, ischemia-reperfusion injury and Parkinson’s disease [95,96,97]. Other materials have also been studied, including H_2_-producing coral calcium hydride that has been shown to reverse brain damage and alleviate oxidative stress and neuroinflammation induced by methamphetamine exposure in mice [98]. The realization of these types of technologies could enable feasible at-home administration of high doses of H_2_. For instance, from a 65 mg Si tablet, more than 100 mL of H_2_ can be generated inside the body, which is equivalent to drinking more than 5 L of H_2_-saturated water.

Hydrogen precursors, for instance Mg or Si particles, can be regarded as hydrogen prodrugs and will, as new chemical entities in drug products, require preclinical and clinical documentation including safety of the materials and the by-products. The in vivo kinetics of H_2_ generation after ingestion will also have to be documented and potentially optimized. Nevertheless, hydrogen therapy holds the potential to emerge as the next breakthrough in the clinical application of nanomedicine. Despite this promise, clinical trials utilizing this cutting-edge technology are yet to be conducted.

The realization of H_2_ therapy’s full potential faces nuanced challenges. Unlike conventional drugs, the medical use of H_2_ cannot directly be protected by intellectual property rights. This might dampen pharmaceutical industry support for clinical trials. However, patenting technology for H_2_ production devices, such as inhalation devices, and new delivery systems is possible. Therefore, the development of these technologies might be necessary for realizing the clinical potential of H_2_ therapy.

## 8. Conclusions

In summary, we are confident that H_2_ therapy will gain recognition as a viable therapeutic treatment approach in the future, for some specific diseases and conditions. However, several of the performed clinical studies might be regarded as anecdotal clinical studies, and future recommended clinical use of hydrogen for treatment of a specific indication might require comparative, double blinded, randomized clinical studies according to guidelines for clinical documentation of new drug substances. Future recommended clinical use of hydrogen requires medical quality of hydrogen gas. Additionally, the medical use of hydrogen requires safe, efficient and practical ways for administration to the patient which might be realized with technological innovations, for instance within nanomedicine.

## Figures and Tables

**Table 1:** Clinical trials studying the safety and therapeutic effects of H_2_ administration registered at clinicaltrials.gov, outlining disease studied, administration method, trial status, the date the trial was first submitted and location. NA = not applicable.

NCT No.	Indication/Condition/Disease	Administration Method	Phase/Status	Date First Posted/Location
	**COVID-19**			
**04594460**	Convalescent COVID-19	Hydrogen-oxygen mixed gas inhalation (2:1, H_2_:O_2_)	NA/ Not yet recruiting	October 2020/China
**04378712**	COVID-19	Hydrogen-oxygen mixed gas inhalation (2:1, H_2_:O_2_)	NA/Completed	May 2020/China
**04336462**	COVID-19	Hydrogen-oxygen mixed gas inhalation (2:1, H_2_:O_2_)	NA/Recruiting	April 2020/China
**05504460**	Discharged patients previously hospitalized for COVID-19 pneumonia	Hydrogen-Oxygen Generator with Nebulizer (inhalation)	NA/Recruiting	August 2022/China
**05539664**	COVID-19	Hydrogen-Oxygen Generator with Nebulizer (inhalation)	NA/Active, not recruiting	August 2022/China
**05770206**	COVID-19	Hydrogen-Oxygen Generator with Nebulizer (inhalation)	NA/Not yet recruiting	February 2023/China
**04633980**	Moderate COVID-19	Inhalation of hydrogen gas 3.6% in N_2_	1/Not yet recruiting	November 2020/France
**04716985**	COVID-19 Patients Treated in Ambulatory Care	Hydrogen-rich water, oral (from dissolved 80 mg magnesium in water), 0.5 L/day	NA/Recruiting	January 2021/Serbia, France and Morocco
	**Lifestyle-related and exercise**			
**03846141**	Health and Exercise Performance (HIHEP)	Hydrogen inhalation, 4%	NA/Completed	February 2019/Serbia
**05842993**	Diabetes type 2	Hydrogen inhalation, 2 L/min	NA/Recruiting	April 2023/China
**05905588**	Diabetes type 2	Hydrogen-rich water, oral	NA/Recruiting	May 2023/China
**05799911**	Exercise performance in professional athletes	Hydrogen-rich water, oral (1–3 L/day)	NA/Completed	March 2023/Czechia
**05862987**	Acute Body Response and Recovery After 5 km Run	Hydrogen-rich water, oral	NA/Enrolling by invitation	May 2023/Czechia
**02832219**	Metabolic fitness in obesity	Hydrogen tablet, oral	3/Completed	July 2016/Serbia
**01759498**	Sport-related Soft Tissue Injuries	Oral hydrogen-rich capsules and/or topical hydrogen-rick packs	2/Completed	January 2013/Serbia
**04167202**	Acute Ankle Sprain	Ankle baths with hydrogen-rich water	NA/Completed	November 2019/Serbia
	**Cancer**			
**03818347**	Cancer rehabilitation	Hydrogen-oxygen mixed gas inhalation (2:1, H_2_:O_2_, 3 L/min)	NA/Completed	January 2019/China
**05728112**	Head and Neck Cancer, Fatigue, Pain and Quality of Life	Hydrogen inhalation	NA/Recruiting	January 2023/Taiwan
**04175301**	Concurrent radiotherapy and chemotherapy for High Grade Glioma patients	Hydrogen-rich water, oral (from dissolved 80 mg magnesium in water)	2/Recruiting	November 2019/NY USA
**04713332**	Radiation-Induced Adverse Events, Rectal Cancer	Hydrogen-rich water, oral (comparing to vitamin E)	3/Recruiting	January 2021/Jordan
**05913895**	Oral Mucositis after Therapy (radiation or combined chemo) in Head and Neck Cancer patients	Hydrogen-rich water, oral	NA/Not yet recruiting	June 2023/Taiwan
**05278260**	Mucositis from Radiation Therapy in Head and Neck Cancer Patients	Hydrogen-rich water, oral	NA/recruiting	March 2022/NY USA
	**Respiratory**			
**04000451**	Acute Exacerbations of Chronic Obstructive Pulmonary Disease (COPD)	Hydrogen-oxygen mixed gas inhalation (2:1, H_2_:O_2_)	NA/Completed	June 2019/China
**02765295**	Bronchiectasis	Hydrogen-oxygen mixed gas inhalation (2:1, H_2_:O_2_)	NA/Recruiting	May 2016/China
**02850185**	Severe COPD Patients	Hydrogen-oxygen mixed gas inhalation (2:1, H_2_:O_2_), adjuvant therapy	NA/Recrutiting	July 2016/China
**02883582**	Severe Asthma	Hydrogen-oxygen mixed gas inhalation (2:1, H_2_:O_2_)	NA/Unknown	August 2016/China
**02961387**	Dyspnea in patients with Tracheal Stenosis	Inhalation of hydrogen gas.	NA/Unknown	November 2016/China
	**CNS and cognitive**			
**02830854**	Cognitive Function and Performance in Elderly	Hydrogen inhalation 3%	3/Completed	July 2016/Serbia
**05891938**	Alzheimer’s disease	Hydrogen inhalation	NA/Completed	May 2023/Korea
**03971617**	Parkinson’s disease	Hydrogen-rich water, oral (from dissolved 80 mg magnesium in water)	2 and 3/Recruiting	June 2019/NY USA
**03320018**	Acute Ischemic Stroke	Oral or IV H_2_ rich solution combined with oral or IV administration of minocycline	2 and 3/Unknown	October 2017/NY USA
	**Autoimmune**			
**05116215**	Autoimmune disease, rheumatologic patients	Either H_2_ capsules, oral, H_2_ inhalation 2% or H_2_-rich water, oral, with conventional treatment	1/Recruiting	October 2021/Taiwan
**02918188**	Chronic graft-versus-host disease	Hydrogen-rich water, oral	2/Recruiting	September 2016/China
**05196295**	Rheumatologic and Metabolic Patients	Hydrogen capsules, oral	1 /Recruiting	January 2022/Taiwan
	**Cardiovascular**			
**05282836**	Aneurysmal Subarachnoid Hemorrhage (HOMA)	Hydrogen-oxygen mixed gas inhalation (2:1, H_2_:O_2_, 3 L/min)	NA/Not yet recruiting	August 2022/China
**05574296**	Cardiac arrest requiring extracorporeal cardiopulmonary resuscitation (ECPR)	Hydrogen gas inhalation 2.4% in medical air	1/Not yet recruiting	October 2022/MA USA
	**Liver**			
**05325398**	Non-Alcoholic Fatty Liver disease	Hydrogen-rich water, oral	NA/Completed	May 2021/Slovakia
**03625362**	Non-Alcoholic Fatty Liver disease	Hydrogen-rich water, oral	NA/Completed	August 2018/Serbia
	**Fatigue**			
**05013606**	Chronic Fatigue Syndrome	Hydrogen-rich water, oral	NA/Completed	February 2019/NY USA
**05397626**	Chronic Fatigue Syndrome	Hydrogen-rich water, oral, with or without HRV-BF (biofeedback)	1/Recruiting	May 2021/NY USA
	**Other**			
**05248360**	Insomnia	Hydrogen-oxygen mixed gas inhalation (2:1, H_2_:O_2_, 900 mL/min)	NA/Enrolling by invitation	February 2022/China
**05476575**	Post-operative Pain and Inflammation Cytokines	4% Hydrogen inhalation via nasal cannula perioperatively	NA/Recruiting	October 2021/Taiwan
**04046211**	Safety of Inhaled Hydrogen Gas Mixtures in Healthy Volunteers	Hydrogen gas inhalation 2.4% in medical air	1/Completed	August 2019/MA USA
**04881435**	Sudden Sensorineural Hearing Loss	Hydrogen gas inhalation with standard steroid treatment	NA/Unknown status	May 2021/Taiwan
**04430803**	Aging	Hydrogen-rich water, oral	NA/Active, not recruiting	June 2020/Serbia
**05556252**	Premenstrual Symptoms and Quality of Life in students with premenstrual syndrome	Hydrogen-rich water, oral	NA/Completed	September 2022/Turkey
**02613195**	Aging grafts in (DBD) liver/kidney transplantation (HRCSDBD)	Hydrogen bath, liver grafts lavaged and cold stored with hydrogen-rich Celsior solution	3/Unknown	November 2015/China

**Table 2:** Clinical trials studying safety and therapeutic effects of H_2_ administration conducted in Japan and registered at umin.ac.jp/ctr, outlining disease studied, administration method and the date of disclosure of the study information.

UMIN ID	Indication/Disease/Effects	Administration Method	Date Posted
	**Lifestyle-related and exercise**		
**000023116**	Body fat-reducing, anti-oxidant and anti-fatigue	Hydrogen-rich water, oral	December 2017
**000029322**	Pre-metabolic syndrome	Hydrogen-rich water, oral	September 2017
**000029321**	Metabolic syndrome	Hydrogen-rich water, oral	September 2017
**000019032**	Type 2 diabetes	Hydrogen-rich water, oral	October 2015
**000018182**	Type 2 diabetes	Hydrogen-rich water, oral	July 2015
**000029062**	Exercise tolerance and fatigue	Hydrogen-rich water, oral	September 2017
**000050872**	Anti-fatigue during exercise	Hydrogen-rich water, oral	April 2023
	**Cancer**		
**000035864**	Side effect in cancer patients receiving radiotherapy	Hydrogen gas inhalation	February 2019
	**CNS and cognitive**		
**000019820**	Neurological outcome following brain ischemia during post-cardiac arrest care	2% hydrogen gas inhalation	January 2016
**000019082**	Parkinson’s disease	3.5% hydrogen gas inhalation	September 2015
**000010014**	Parkinson’s disease	Hydrogen-rich water, oral	February 2013
**000007497**	Parkinson’s disease	Hydrogen-rich water, oral	March 2012
**000008959**	Multiple system atrophy and Progressive supranuclear palsy	Hydrogen-rich water, oral	October 2012
	**Cardiovascular**		
**000014630**	Ischemia-reperfusion after lung transplantation	1.3% hydrogen gas inhalation	August 2014
**000014390**	Acute myocardial infarction	Hydrogen gas inhalation	July 2014
**000032523**	Peripheral endothelial function	Hydrogen-rich water, oral	May 2018
**000032510**	Peripheral endothelial function	Hydrogen-rich water, oral	May 2018
**000033459**	Peripheral endothelial function	Hydrogen-rich water, oral	July 2018
**000032701**	Cardiovascular diseases	Hydrogen tablet, oral	May 2019
**000021154**	Hypertension	Hydrogen tablet, oral	February 2016
	**Liver**		
**000010693**	Chronic hepatitis and liver cirrhosis	Hydrogen-rich water, oral	May 2013
	**Fatigue**		
**000027700**	Anti-fatigue	Hydrogen-rich water, oral	November 2018
	**Safety and distribution**		
**000023550**	H_2_ concentrations in expired air after intake of hydrogen-rich water and water containing indigestible sugars	Hydrogen-rich water, oral	August 2016
**000037169**	Change in H_2_ concentration in breath by H_2_ supplementation	Hydrogen-rich water, oral	June 2019
**000036250**	Change in H_2_ concentration in breath by H_2_ supplementation	Hydrogen-rich water, oral	March 2019
**000013221**	Safety of hydrogen eye drop	Hydrogen eye drop	February 2014
	**Physiological/molecular markers**		
**000051588**	Effects of hydrogen gas inhalation on oxidized lipids	Hydrogen gas inhalation by nasal inhaler	July 2023
**000005779**	Detection of reduction in oxidative stress	Hydrogen-rich water, oral	September 2011
**000039708**	Effects of H_2_ on the cytokine and oxidative stress levels in pregnant women	Hydrogen-rich water, oral	April 2020
**000019654**	Effects of H_2_ water on gut peptide	Hydrogen-rich water, oral	November 2015
**000033102**	Effects of H_2_ water on gut microbiota	Hydrogen-rich water, oral	June 2018
	**Other**		
**000033110**	Pain/swelling of the hands and arms	Hydrogen-rich water bath	June 2018
**000015528**	Ischemia-reperfusion injury of retinal artery occlusion	Hydrogen-rich eye drop	October 2014
**000045459**	Systemic inflammatory reaction syndrome	Injection of H_2_-rich water using a catheter-tipped syringe directly from the gastric tube	September 2021

## Data Availability

Data will be made available on request.
